# Utility of urinary presepsin in the diagnosis of pyelonephritis: a cross-sectional study

**DOI:** 10.1186/s12879-023-08353-2

**Published:** 2023-05-31

**Authors:** Ryo Yamashita, Yusuke Izumi, Koji Nakada, Jun Hiramoto

**Affiliations:** 1 Department of General Medicine, The Jikei University School of Medicine, Daisan Hospital, 4-11-1 Izumihoncho, Komae-Si, Tokyo 201-0003 Japan; 2 Department of Laboratory Medicine, The Jikei University School of Medicine, Daisan Hospital, 4-11-1 Izumihoncho, Komae-Si, Tokyo 201-0003 Japan

**Keywords:** Presepsin, Pyelonephritis, Pyuria, Receiver operating characteristics

## Abstract

**Background:**

Presepsin is produced during the phagocytosis of bacteria by granulocytes. Presepsin increases at the site of infection; however, the significance of urinary presepsin in pyelonephritis is unknown. This study aimed to evaluate whether measuring urinary presepsin can distinguish between pyelonephritis and nonpyelonephritis.

**Methods:**

A cross-sectional study of patients with suspected pyelonephritis was conducted. Urinary presepsin at admission was compared between the pyelonephritis and nonpyelonephritis groups using the Mann–Whitney test. The predictive accuracy of urinary presepsin for diagnosing pyelonephritis was evaluated by the area under the receiver operating characteristics (ROC) analysis curve.

**Results:**

A total of 35 eligible participants were included in the pyelonephritis group and 25 in the nonpyelonephritis group. The median urinary presepsin level was 2232.0 (interquartile range [IQR], 1029.0–3907.0) pg/mL in the pyelonephritis group and 1348.0 (IQR, 614.5–2304.8) pg/mL in the nonpyelonephritis group. Urinary presepsin concentrations were significantly higher in the pyelonephritis group than in the nonpyelonephritis group (*P* = 0.023). ROC analysis of urinary presepsin revealed a cutoff value of 3650 pg/mL to distinguish between the pyelonephritis and nonpyelonephritis groups. Sensitivity, specificity, positive predictive value, negative predictive value, positive likelihood ratio, and negative likelihood ratio for the diagnosis of pyelonephritis were 0.40 (95% confidence interval [CI], 0.24–0.58), 0.96 (95% CI, 0.79–1.00), 0.93 (95% CI, 0.68–1.00), 0.52 (95% CI, 0.37–0.68), 9.60 (95% CI, 1.35–68.23), and 0.62 (95% CI, 0.47–0.83), respectively.

**Conclusions:**

The measurement of urinary presepsin is useful in differentiating pyelonephritis from other diseases.

**Supplementary Information:**

The online version contains supplementary material available at 10.1186/s12879-023-08353-2.

## Background

Pyelonephritis is a common disease that is more likely to occur in young women and older individuals. The symptoms and severity of the disease range widely. In most patients, the symptoms are mild back pain and fever, but septic shock can also occur [1]. If severe sepsis or septic shock occurs, the mortality rate is reported to be 20%–50% [2, 3]. Accurate diagnosis and early treatment are important. Although there is no consensus on the diagnostic criteria for pyelonephritis [4], detecting bacteria in urine culture is crucial. However, obtaining the results of urine culture can take several days; hence, they are not useful for the early diagnosis of pyelonephritis. In addition, the results of urine culture depend on proper specimen collection; however, improper specimen collection can lead to false-positive results. Pyuria is another characteristic finding of pyelonephritis and can be detected early. However, pyuria can also be detected in asymptomatic bacteriuria. Hence, the presence of pyuria does not prove pyelonephritis. There are no tools that can quickly distinguish between pyelonephritis and nonpyelonephritis. New biomarkers which can accurately and rapidly diagnose pyelonephritis are needed. Presepsin, a granulocyte degradation product, is produced when granulocytes phagocytose bacteria [5]. Presepsin has been attracting attention as a useful marker for diagnosis and prognosis in sepsis [6]. Since presepsin production is increased at the site of infection, its measurement in the joint fluid is effective in differentiating pyogenic arthritis from crystalline arthritis [7]. The cerebrospinal fluid presepsin is also useful for the diagnosis and prognosis of post-neurosurgical ventriculitis/meningitis [8]. The significance of urinary presepsin in diagnosing pyelonephritis is unknown. This study aimed to evaluate whether measuring urinary presepsin can distinguish between pyelonephritis and nonpyelonephritis.

## Methods

### Study design and participants

This was a cross-sectional study performed on 132 patients admitted to Jikei University Daisan Hospital with suspected pyelonephritis from October 2019 to November 2021. The inclusion criteria for the study were (1) patients over 20 years, (2) patients with a fever of 37.5 °C or higher, (3) and patients with a urine sedimentation test showing over 10 leukocytes/high power field. Acute pyelonephritis was defined as a patient having back pain or costovertebral angle tenderness and urine culture revealing a bacterial count of 10^5^/liter or higher. We used midstream urine for urine culture. Patients not meeting the definition of acute pyelonephritis were defined as the nonpyelonephritis group. The exclusion criteria for the study were (1) patients receiving antibiotics within a week before admission, (2) patients with indwelling urinary catheters, (3) patients with anatomical abnormalities in the urinary tract, and (4) patients on dialysis.

### Study measurements

The patients’ age, gender, body temperature, past medical history, and the results of blood and urine cultures at the time of admission were collected. All patients underwent blood sampling for serum C-reactive protein, procalcitonin, creatinine, and presepsin on admission day. Urinary presepsin was also measured on admission day. Serum and urinary presepsin concentrations were measured by a PathFast immunoanalyzer presepsin kit (LSI Medicine, Tokyo, Japan).

### Statistical analysis

The Bell curve for Excel (Social Survey Research Information Co., Ltd., Tokyo, Japan) was used for all the statistical analyses. The Mann–Whitney U test was used to compare the age, urinary presepsin, serum C-reactive protein, procalcitonin, creatinine, estimated glomerular filtration rate, and serum presepsin. The chi-square test was used to compare the sex, past medical history, and bacteremia. The predictive accuracy of urinary presepsin and serum presepsin for diagnosing acute pyelonephritis was evaluated by the area under the curve (AUC) of ROC analysis. The Youden index was used to calculate optimal cutoff values for each measurement result. The cutoff values were used to calculate the sensitivity, specificity, positive predictive value (PPV), negative predictive value (NPV), positive likelihood ratio (LR +), and negative likelihood ratio (LR −) for each measure. The *P*-values of < 0.05 were considered statistically significant.

## Results

### Baseline characteristics of participants

Among the 132 participants who met the inclusion criteria, 60 participants were included in this study (41 participants were post-antibiotic, 11 participants had indwelling catheters, 18 participants had anatomical abnormalities in the urinary tract, and two participants were on dialysis). The participants were divided into two groups: acute pyelonephritis (*n* = 35) and nonpyelonephritis (*n* = 25). The average age of all participants was 77.6 years, and 78.3% of the participants were female patients. There were no significant differences between the two groups for age, sex, background disease, or immunosuppressive drug use. The bacteremia rate was significantly higher in the acute pyelonephritis group (*P* = 0.044). There were also no significant differences in the renal function, serum C-reactive protein, presepsin, or procalcitonin between the acute pyelonephritis and nonpyelonephritis groups (Table 1).

**Table 1:** Baseline characteristics of participants

	**Acute pyelonephritis** (*n* = 35)	**Nonpyelonephritis** (*n* = 25)	***P*-value**
Age (years)	85 [78.0–90.5]	81 [56.0–88.0]	0.064
Female sex	25 (71)	22 (88)	0.125
Diabetes mellitus	9 (26)	4 (16)	0.368
Liver cirrhosis	0 (0)	1 (4.0)	0.233
Chronic heart failure	3 (8.6)	2 (8.0)	0.937
Cerebrovascular disorder	9 (26)	2 (8.0)	0.080
Malignant tumor	3 (8.6)	3 (12)	0.663
Autoimmune disease	8 (23)	5 (20)	0.791
Immunosuppressor	7 (20)	3 (12)	0.412
Bacteremia	8 (23)	1 (4.0)	0.044
Creatinine (mg/dL)	0.90 [0.68–1.25]	0.84 [0.51–1.07]	0.161
eGFR (mL/min/1.73 m^2^)	49.0 [38.0–66.5]	64.0 [42.0–100.0]	0.122
C-reactive protein (mg/L)	113.4 [50.3–194.1]	88.9 [29.0–148.9]	0.393
Serum Presepsin (pg/mL)	361 [302–589]	386 [251–650]	0.844
Procalcitonin (ng/mL)	0.38 [0.17–2.06]	0.19 [0.08–0.94]	0.097

Values are expressed as number (%) or median [interquartile range, IQR]

Abbreviations: *eGFR* estimated glomerular filtration rate

The final diagnosis in the non-pyelonephritis group is shown in Table 2.

**Table 2:** Final diagnosis in the nonpyelonephritis group

Diagnosis	Number of cases (%)
Pneumonia	11 (44)
Enterocolitis	2 (8)
Crystal arthritis	2 (8)
Giant cell arthritis	1 (4)
Microscopic polyangiitis	1 (4)
Adult-onset Still's disease	1 (4)
Viral meningitis	1 (4)
Cholangitis	1 (4)
Histiocytic necrotizing lymphadenitis	1 (4)
Hemophagocytic lymphohistiocytosis	1 (4)
Hematoma	1 (4)
Ovarian hemorrhage	1 (4)
Drug fever	1 (4)

### Comparison of urinary presepsin concentrations in acute pyelonephritis and nonpyelonephritis groups

**Fig. 1:**
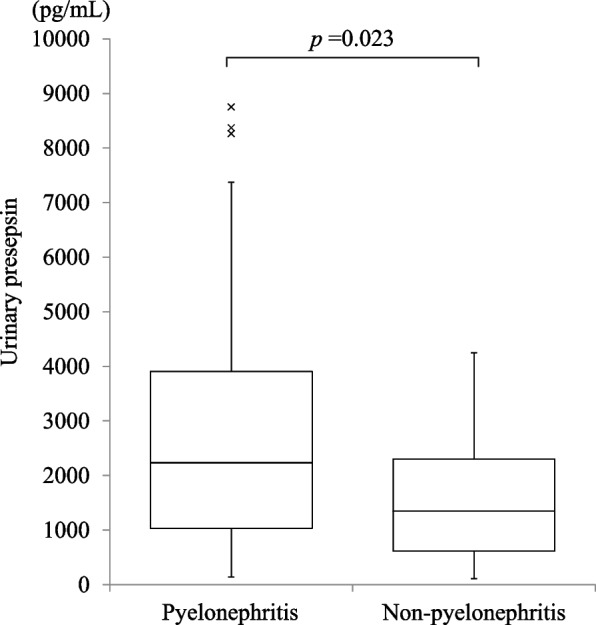
Comparison of urinary presepsin levels in patients with pyelonephritis and nonpyelonephritis. The Mann–Whitney U test was used for comparing the two groups. Urinary presepsin concentrations were significantly higher in the acute pyelonephritis group than in the nonpyelonephritis group

The median urinary presepsin level was 2232.0 (interquartile range [IQR], 1029.0–3907.0) pg/mL in the acute pyelonephritis group and 1348.0 (IQR, 614.5–2304.8) pg/mL in the nonpyelonephritis group. Urinary presepsin concentrations were significantly higher in the acute pyelonephritis group than in the nonpyelonephritis group (*P* = 0.023) (Fig. 1).

### Comparison of urinary presepsin levels between patients with and without bacteremia among those with acute pyelonephritis

The median urinary presepsin level was 2463.0 pg/mL (IQR, 1252.8–7215.8) in patients with acute pyelonephritis with bacteremia and 2385.0 pg/mL (IQR, 1138.0–4043.5) in patients with acute pyelonephritis without bacteremia. Urinary presepsin levels were not significantly different between the two groups (*P* = 0.875) (sTable 1).

### Comparison of urinary presepsin levels between the pyelonephritis and nonpyelonephritis groups among patients without bacteremia

The median urinary presepsin level was 2385.0 pg/mL (IQR, 1138.0–4043.5) in the acute pyelonephritis group without bacteremia and 1339.0 pg/mL (IQR, 594.0–2350.5) in the nonpyelonephritis group without bacteremia. Urinary presepsin was significantly higher in the acute pyelonephritis group without bacteremia than in the nonpyelonephritis group without bacteremia (*P* = 0.026) (sTable 2).

### The accuracy of urinary presepsin in the diagnosis of acute pyelonephritis

**Fig. 2:**
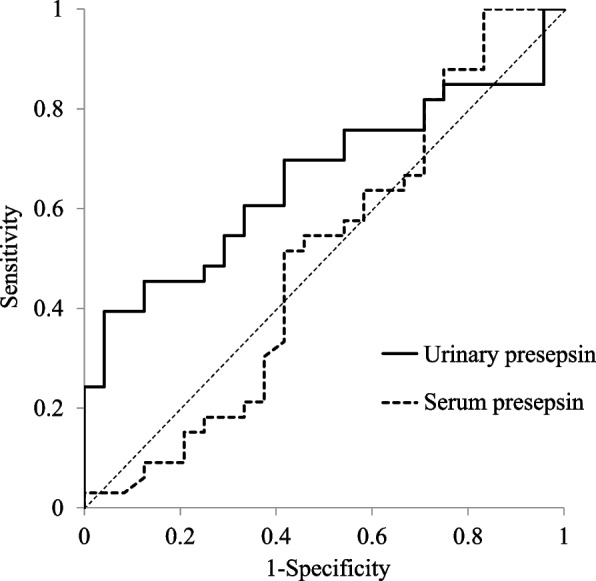
ROC curves for the sensitivity and specificity of urinary presepsin and serum presepsin in differentiating pyelonephritis from other diseases. The value of AUC was 0.66 (95% confidence interval [CI], 0.52–0.80)

ROC curve analysis of urinary presepsin revealed a cutoff value of 3650 pg/mL to distinguish the acute pyelonephritis and nonpyelonephritis groups (Fig. 2). The value of AUC was 0.66 (95% confidence interval [CI], 0.52–0.80). Using the calculated cutoff values, sensitivity, specificity, PPV, NPV, LR + , and LR − for the diagnosis of acute pyelonephritis were 0.40 (95% CI, 0.24–0.58), 0.96 (95% CI, 0.79–1.00), 0.93 (95% CI, 0.68–1.00), 0.52 (95% CI, 0.37–0.68), 9.60 (95% CI, 1.35–68.23), and 0.62 (95% CI, 0.47–0.83), respectively. Compared to serum presepsin, urinary presepsin was a more accurate indicator of sensitivity, specificity, PPV, NPV, LR + , and LR − (Table 3).

**Table 3:** Ability of urinary presepsin and serum presepsin to detect acute pyelonephritis

	**AUC**	**Cutoff**	**Sensitivity**	**Specificity**	**PPV**	**NPV**	**LR + **	**LR-**
Urinary presepsin	0.66 [0.52–0.80]	3650 pg/mL	0.40 [0.24–0.58]	0.96 [0.79–1.00]	0.93 [0.68–1.00]	0.52 [0.37–0.68]	9.60 [1.35–68.23]	0.62 [0.47–0.83]
Serum presepsin	0.50 [0.34–0.66]	1522 pg/mL	0.03 [0.00–0.16]	0.84 [0.64–0.95]	0.20 [0.01–0.72]	0.40 [0.26–0.54]	0.19 [0.02–1.59]	1.15 [0.96–1.38]

Results are expressed as values [95% confidence interval]

Abbreviations: *AUC* Area under the ROC curve, *PPV* Positive predictive value, *NPV* Negative predictive value, *LR* + Positive likelihood ratio, *LR − *Negative likelihood ratio

## Discussion

This study revealed that urinary presepsin was significantly elevated in the acute pyelonephritis group compared with the nonpyelonephritis group. On the contrary, serum CRP, procalcitonin, and presepsin were not significantly different between the pyelonephritis and the nonpyelonephritis groups. These results suggest that measuring urinary presepsin in patients with fever and pyuria may provide an early indication of pyelonephritis.

When granulocytes phagocytose bacteria, CD14 is taken into the cells and degraded by proteases. The degraded CD14 becomes soluble CD14, which is detected as presepsin [5]. Presepsin is one of the biomarkers elevated in bacterial infections because of this production mechanism [9]. In pyelonephritis with bacteremia, serum presepsin levels are increased [10]. Furthermore, serum presepsin concentration correlates with sepsis severity. It is a more useful biomarker than PCT in predicting disease severity [11]. In pyelonephritis, granulocytes phagocytose bacteria in the urinary tract. This may result in increased local presepsin production and high urinary presepsin levels. For diagnosing pyelonephritis, urine culture results need to be awaited. However, it takes several days to obtain the urine culture results. On the contrary, urinary presepsin can be measured in 15 min. Although the definitive diagnosis of pyelonephritis is based on the identification of bacteria by urine culture, it is undoubtedly important to base antibiotic selection on the results of antibiotic sensitivity testing. Urinary presepsin cannot replace this significant utility of urine culture. However, it may contribute to an earlier diagnosis of pyelonephritis, which may help in reducing unnecessary antibiotic usage.

Our additional analyses revealed no significant difference in urinary presepsin levels between patients with and without bacteremia among those with pyelonephritis. This finding might be due to the fact that urinary presepsin mainly reflects local presepsin production in the urinary tract rather than the effects of bacteremia. In the present study, the comparison of patients with pyelonephritis in the absence of bacteremia and those without pyelonephritis or bacteremia revealed that urinary presepsin levels were significantly high in those with pyelonephritis without bacteremia, suggesting that the higher urinary presepsin levels observed in patients with pyelonephritis, as shown in Fig. 1, was not due to a higher percentage of patients with bacteremia.

The results of ROC curve analysis revealed that urinary presepsin has higher sensitivity, specificity, PPV, NPV, LR + , and LR − than serum presepsin in the diagnosis of acute pyelonephritis. Unfortunately, the AUC and sensitivity of urinary presepsin were not high. Therefore, combining urinary presepsin with other laboratory parameters might be more useful for the diagnosis of acute pyelonephritis. Traditionally, the assessment for acute pyelonephritis is often performed using leukocyte esterase, an enzyme released by leukocytes, and nitrites as some bacteria reduce urinary nitrates to nitrites. When either leukocyte elastase or nitrite is positive, a diagnosis of acute pyelonephritis can be made with a sensitivity of 75%, specificity of 82%, LR + of 4.2, and LR − of 0.3 [12]. Although urinary presepsin is inferior to the combination of leukocyte elastase and nitrites in sensitivity, it may be superior in specificity, LR + , and LR − . Urinary presepsin may be a useful test for making the definitive diagnosis of acute pyelonephritis. For pyelonephritis, we recommend that urinalysis should be initially performed to screen for leukocyte elastase and nitrite and that urinary presepsin levels should be measured in patients with a positive screening result to get closer to the definitive diagnosis.

This study has several limitations. First, the sample size was small because the patients with factors that might affect urinary presepsin levels were excluded from this study. To clarify the generalizability of the results, further investigations are required. Second, this study included patients with fever and pyuria. Patients without fever were not included in this study. In addition, severe cases and older patients with pyelonephritis not presenting with fever were not included in this study. Hence, additional studies are needed. Third, patients with catheter-related urinary tract infections, anatomical abnormalities of the urinary tract, and hemodialysis patients were excluded from this study. It is unclear whether urinary presepsin measurement would be useful in these patients. Fourth, some of the patients in the nonpyelonephritis group might have had cystitis or asymptomatic bacteriuria. To the best of our knowledge, there are no studies comparing urinary presepsin levels between pyelonephritis and other urinary tract infections such as cystitis or asymptomatic bacteriuria, and it is unclear whether these conditions affect urinary presepsin. Further studies are needed to elucidate this aspect. Finally, this study was conducted with a single-center design. Further investigations at other facilities are required.

## Conclusion

In conclusion, urinary presepsin measurement is useful in differentiating pyelonephritis from other diseases. We have shown that urinary presepsin has high specificity, PPV, and LR + in the diagnosis of pyelonephritis. Urinary presepsin provides immediate results and may contribute to appropriate antibiotic use.

## Supplementary Information

**Additional file 1**: **sTable 1.** Comparison in pyelonephritis group with and without bacteremia. **sTable 2.** Comparison of pyelonephritis and nonpyelonephritis groups in patients with negative blood cultures.

## Data Availability

The used and/or analyzed data during the current study is available from the corresponding author on reasonable request.

## Abbreviations

AUC: Area under the curve
LR: Likelihood ratio
NPV: Negative predictive value
PPV: Positive predictive value
ROC: Receiver operating characteristics

## References

[1] James JR, Russo TA (2018). Acute pyelonephritis in adults. N Engl J Med.

[2] Angus DC, Linde-Zwirble WT, Lidicker J, Clermont G, Carcillo J, Pinsky MR (2001). Epidemiology of severe sepsis in the United States: analysis of incidence, outcome, and associated costs of care. Crit Care Med.

[3] Levy MM, Artigas A, Phillips GS, Rhodes A, Beale R, Osborn T (2012). Outcomes of the surviving sepsis campaign in intensive care units in the USA and Europe: a prospective cohort study. Lancet Infect Dis.

[4] Piccoli GB, Consiglio V, Colla L, Mesiano P, Magnano A, Burdese M (2006). Antibiotic treatment for acute 'uncomplicated' or 'primary' pyelonephritis: a systematic, 'semantic revision'. Int J Antimicrob Agents.

[5] Arai Y, Mizugishi K, Nonomura K, Naitoh K, Takaori-Kondo A, Yamashita K (2015). Phagocytosis by human monocytes is required for the secretion of presepsin. J Infect Chemother.

[6] Lee S, Song J, Park DW, Seok H, Ahn S, Kim J (2022). Diagnostic and prognostic value of presepsin and procalcitonin in noninfectious organ failure, sepsis, and septic shock: a prospective observational study according to the Sepsis-3 definitions. BMC Infect Dis.

[7] Imagama T, Seki K, Seki T, Tokushige A, Matsuki Y, Yamazaki K (2021). Synovial fluid presepsin as a novel biomarker for the rapid differential diagnosis of native joint septic arthritis from crystal arthritis. Int J Infect Dis.

[8] Zheng G, Zhang C, Zhang G, Shao C (2021). Evaluation of the diagnostic and prognostic value of CSF presepsin levels in patients with postneurosurgical ventriculitis/meningitis. Infect Drug Resist.

[9] Memar MY, Baghi HB (2019). Presepsin: a promising biomarker for the detection of bacterial infections. Biomed Pharmacother.

[10] Claessens Y-E, Trabattoni E, Grabar S, Quinquis L, Der Sahakian G, Anselmo M (2017). Plasmatic presepsin (sCD14-ST) concentrations in acute pyelonephritis in adult patients. Clin Chim Acta.

[11] Yaegashi Y, Shirakawa K, Sato N, Suzuki Y, Kojika M, Imai S (2005). Evaluation of a newly identified soluble CD14 subtype as a marker for sepsis. J Infect Chemother.

[12] Bent S, Nallamothu BK, Simel DL, Fihn SD, Saint S (2002). Does this woman have an acute uncomplicated urinary tract infection?. JAMA.

