# Supplementary material for: Utility of urinary presepsin in the diagnosis of pyelonephritis: a cross-sectional study
Source: BMC Infect Dis. 2023 May 31;23:365. doi: 10.1186/s12879-023-08353-2 (PMC10230669; doi:10.1186/s12879-023-08353-2)
Supplement: Supplementary file 1 — Additional file 1: sTable 1. Comparison in pyelonephritis group with and without bacteremia. sTable 2. Comparison of pyelonephritis and nonpyelonephritis groups in patients with negative blood cultures. [file 12879_2023_8353_MOESM1_ESM.docx]

**Utility of urinary presepsin in the diagnosis of pyelonephritis:a cross-sectional study**

Ryo Yamashita^1^, Yusuke Izumi^1^, Koji Nakada^2^, Jun Hiramoto^1^

^1^Department of General Medicine, The Jikei University School of Medicine, Daisan Hospital, 4-11-1 Izumihoncho, Komae-si, Tokyo, 201-0003, Japan

^2^Department of Laboratory Medicine, The Jikei University School of Medicine, Daisan Hospital, 4-11-1 Izumihoncho, Komae-si, Tokyo, 201-0003, Japan

**Correspondence to:**

**Name**: Ryo Yamashita

**Address**: Department of General Medicine, The Jikei University School of Medicine, Daisan Hospital, 4-11-1 Izumihoncho, Komae-si, Tokyo, 201-0003, Japan

Tel: +81-3-3480-1151**;** FAX: +81-3-3430-3611**;**

E-mail: ryoyamashita@jikei.ac.jp

**sTable 1 Comparison** **in pyelonephritis group with and without bacteremia.**

|  | **Bacteremia**  (n = 8) | **Nonbacteremia**  (n = 27) | **P-value** |
| --- | --- | --- | --- |
| Age (years) | 83.5 [71.8–84.3] | 86 [79.5–94] | 0.098 |
| Female sex | 5(63) | 20 (74) | 0.525 |
| Creatinine (mg/dL) | 1.35 [1.07–1.54] | 0.82 [0.64–1.10] | 0.024 |
| eGFR (mL/min/1.73 m^2^) | 32.0 [28.8–42.3] | 50.0 [45.5–70.5] | 0.017 |
| C-reactive protein (mg/L) | 127.5 [63.6–20.7] | 98.3 [33.8–191.5] | 0.271 |
| Procalcitonin (ng/mL) | 3.05 [0.74–10.0] | 0.24 [0.13–0.62] | 0.003 |
| Serum Presepsin (pg/mL) | 613 [356–947] | 352 [302–543] | 0.089 |
| Urinary Presepsin (pg/mL) | 2463 [1253–7216] | 2385 [1138–4044] | 0.875 |
| Values are expressed as number (%) or median [interquartile range, IQR].  Abbreviations: eGFR estimated glomerular filtration rate. | | | |

**sTable 2 Comparison** **of pyelonephritis and nonpyelonephritis groups in patients with negative blood cultures.**

|  | **Pyelonephritis**  (n = 27) | **Nonpyelonephritis**  (n = 24) | **P-value** |
| --- | --- | --- | --- |
| Age (years) | 86 [79.5–94] | 80.5 [52.8–88.5] | 0.026 |
| Female sex | 20 (74) | 21 (88) | 0.228 |
| Creatinine (mg/dL) | 0.82 [0.64–1.10] | 0.79 [0.51–1.08] | 0.485 |
| eGFR (mL/min/1.73 m^2^) | 50.0 [45.5–70.5] | 68.0 [40.3–102.3] | 0.299 |
| C-reactive protein (mg/L) | 98.3 [33.8–191.5] | 93.4 [26.9–149.4] | 0.644 |
| Procalcitonin (ng/mL) | 0.24 [0.13–0.62] | 0.18 [0.08–0.48] | 0.326 |
| Serum Presepsin (pg/mL) | 352 [302–543] | 383 [246–553] | 0.984 |
| Urinary Presepsin (pg/mL) | 2385 [1138–4044] | 1339 [594–2351] | 0.026 |
| Values are expressed as number (%) or median [interquartile range, IQR].  Abbreviations: eGFR estimated glomerular filtration rate. | | | |
